# Paradoxical Effect of Myosteatosis on the Immune Checkpoint Inhibitor Response in Metastatic Renal Cell Carcinoma

**DOI:** 10.1002/jcsm.13758

**Published:** 2025-03-07

**Authors:** Jiwoong Yu, Hyeonju Ahn, Kyung Yeon Han, Wan Song, Hyun Hwan Sung, Hwang Gyun Jeon, Byong Chang Jeong, Seong Il Seo, Seong Soo Jeon, Se Hoon Park, Woong‐Yang Park, Ji Hyun Lee, Minyong Kang

**Affiliations:** ^1^ Department of Urology, Samsung Medical Center, School of Medicine Sungkyunkwan University Seoul South Korea; ^2^ Samsung Genome Institute Samsung Medical Center Seoul South Korea; ^3^ Division of Hematology‐Oncology, Department of Internal Medicine, Samsung Medical Center, School of Medicine Sungkyunkwan University Seoul South Korea; ^4^ Department of Radiology, Samsung Medical Center, School of Medicine Sungkyunkwan University Seoul South Korea; ^5^ Department of Health Sciences and Technology, SAIHST Sungkyunkwan University Seoul South Korea

**Keywords:** body composition, immune checkpoint inhibitors, low skeletal muscle mass, metastatic renal cell carcinoma, myosteatosis, single‐cell RNA sequencing

## Abstract

**Background:**

Treatment for metastatic renal cell carcinoma (mRCC) has shifted from tyrosine kinase inhibitor (TKI) therapy to immune checkpoint inhibitor (ICI)–based therapy, improving outcomes but with variable individual responses. This study investigated the prognostic implications of pretreatment low skeletal muscle mass (LSMM) and myosteatosis in patients with mRCC undergoing first‐line ICI‐based therapies, comparing outcomes between PD‐1 inhibitor + CTLA‐4 inhibitor and PD‐1 inhibitor + TKI, incorporating single‐cell RNA sequencing.

**Methods:**

A retrospective analysis was performed on 90 patients with mRCC treated with ICI‐based therapies between November 2019 and March 2023. Patients were grouped based on whether they received PD‐1 inhibitor + CTLA‐4 inhibitor or PD‐1 inhibitor + TKI combinations. LSMM was defined as skeletal muscle index below 40.8 cm^2^/m^2^ for men and 34.9 cm^2^/m^2^ for women. Myosteatosis was defined using skeletal muscle density, with cut‐off values < 41 HU for BMI < 25 kg/m^2^ and < 33 HU for BMI ≥ 25 kg/m^2^. Progression‐free survival (PFS) and overall survival (OS) were compared using Kaplan–Meier curves and multivariable models. Single‐cell RNA sequencing was performed on pretreatment samples to compare the immune microenvironment between patients with and without myosteatosis.

**Results:**

The study cohort (26.7% female; median age: 60.5 years) included 59 patients (65.6%) treated with PD‐1 inhibitor + CTLA‐4 inhibitor and 31 patients (34.4%) treated with PD‐1 inhibitor + TKI. LSMM was present in 18.9% of patients, and myosteatosis in 41.1%, with comparable proportions across groups. During follow‐up, 29 patients (32.2%) died: 16 in the PD‐1 inhibitor + CTLA‐4 inhibitor group and 13 in the PD‐1 inhibitor + TKI group. The overall 1‐year mortality rate was 22.2%, and PFS rate was 53.3%. Myosteatosis predicted poor OS (HR, 5.389; *p* = 0.008) and PFS (HR, 2.930; *p* = 0.022) in the PD‐1 inhibitor + TKI group but was protective for PFS (HR, 0.461; *p* = 0.049) in the PD‐1 inhibitor + CTLA‐4 inhibitor group. LSMM did not significantly affect outcomes in either group. Single‐cell RNA sequencing revealed higher CTLA‐4 expression in regulatory T cells and more effector memory CD8^+^ T cells in patients with myosteatosis, whereas patients without myosteatosis had more anti‐tumoural non‐classical monocytes.

**Conclusions:**

Myosteatosis negatively impacts OS and PFS in patients with mRCC treated with PD‐1 inhibitor + TKI therapy but is protective for PFS in those treated with PD‐1 inhibitor + CTLA‐4 inhibitor therapy. Altered checkpoint expression and immune cell composition associated with myosteatosis may contribute to these differential responses.

## 1 Introduction

The treatment landscape for metastatic renal cell carcinoma (mRCC) has recently evolved, transitioning from tyrosine kinase inhibitor (TKI) monotherapy towards immune checkpoint inhibitor (ICI)–based therapy, which targets the tumour microenvironment (TME) and enhances anti‐tumour activity by counteracting immune tolerance mechanisms [1]. Although ICI‐based therapy has improved mRCC prognosis, the therapeutic response and duration vary among patients [2]. Therefore, identifying prognostic factors is essential to predict which patient groups are most likely to experience a favourable response. The International Metastatic Renal Cell Carcinoma Database Consortium (IMDC) risk criteria are currently considered the gold standard for predicting the survival of patients with mRCC [3]. However, these criteria, which encompass laboratory values and clinical findings, have limitations, particularly in reflecting host immunity and the TME. Moreover, although programmed death ligand 1 (PD‐L1) expression and tumour mutational burden have been investigated as predictive markers, they have not exhibited significant predictive value, as both the high and low PD‐L1 expression groups experience similar benefits from checkpoint inhibitors. Moreover, tumour mutational burden has not been identified as a predictive parameter [S1, S2].

Body composition has emerged as an important prognostic factor in cancer patients, particularly in mRCC. Researchers have increasingly focused on pretreatment body composition measurements, especially muscle‐related metrics. Computed tomography (CT) imaging offers the advantage of objectively measuring the quantity and quality of muscle mass. These parameters reflect nutritional deficiencies, metabolic dysregulation and immune function, making them potential prognostic indicators for ICI‐based therapy efficacy in mRCC [4, 5, 6]. In particular, pretreatment low skeletal muscle mass (LSMM), defined by a decline in skeletal muscle mass as measured by muscle area, has been extensively studied. However, evidence regarding its correlation with the clinical outcomes in patients with mRCC undergoing ICI‐based therapy remains conflicting [4, 5]. Furthermore, myosteatosis, characterized by reduced muscle density due to increased intramuscular fat, has been linked to unfavourable clinical responses to ICI‐based therapy [6]. Moreover, chronic inflammation triggered by cancer and the associated immune response induces the infiltration and activation of various immune cells in the skeletal muscle, impacting muscle catabolic pathways [7]. Thus, cancer‐induced chronic inflammation has a radiologically discernible impact on muscles [8, 9]. The relationship between myosteatosis and ICI‐based therapy has been investigated across various cancer types, including kidney, lung, liver and urothelial cancers [S3–S5].

However, current studies have predominantly focused on evaluating these muscle‐related parameters in patients receiving ICI‐based therapy beyond the first‐line setting, either following TKI therapy or in combination regimens such as PD‐1 inhibitor + TKI or PD‐1 inhibitor + CTLA‐4 inhibitor therapy. The administration of TKI, either before or concurrently with ICI, can exert distinct biological effects on fat and muscle metabolism, including considerable muscle deterioration [10, 11, 12]. Additionally, reports have suggested differences in the prognostic role of body composition metrics between the use of ICI‐based therapy in first‐line versus later‐line treatments [13, 14]. This complexity makes it challenging to fully understand the influence of these muscle‐related parameters on responses to ICI‐based therapy. Accordingly, in the current study, we sought to investigate the prognostic implications of pretreatment LSMM and myosteatosis in the context of first‐line ICI‐based therapy. To achieve this, we conducted a retrospective comparative analysis between patients receiving PD‐1 inhibitor + CTLA‐4 inhibitor therapy and those receiving PD‐1 inhibitor + TKI therapy for mRCC. Furthermore, we analysed single‐cell mRNA levels to elucidate the relationships between muscle‐related parameters and patient prognosis.

## 2 Materials and Methods

### 2.1 Patients and Data

This study was approved by the Institutional Review Board of our institution (IRB No. 2023‐12‐089 and 2020‐03‐063). Due to its retrospective nature involving a review of medical records, the requirement for informed consent was waived. However, written informed consent was obtained from patients who provided tissue samples for single‐cell RNA sequencing (scRNA‐seq). All study procedures were conducted in accordance with the Declaration of Helsinki, and all patient data were handled in compliance with relevant privacy regulations and data protection laws.

**FIGURE 1:**
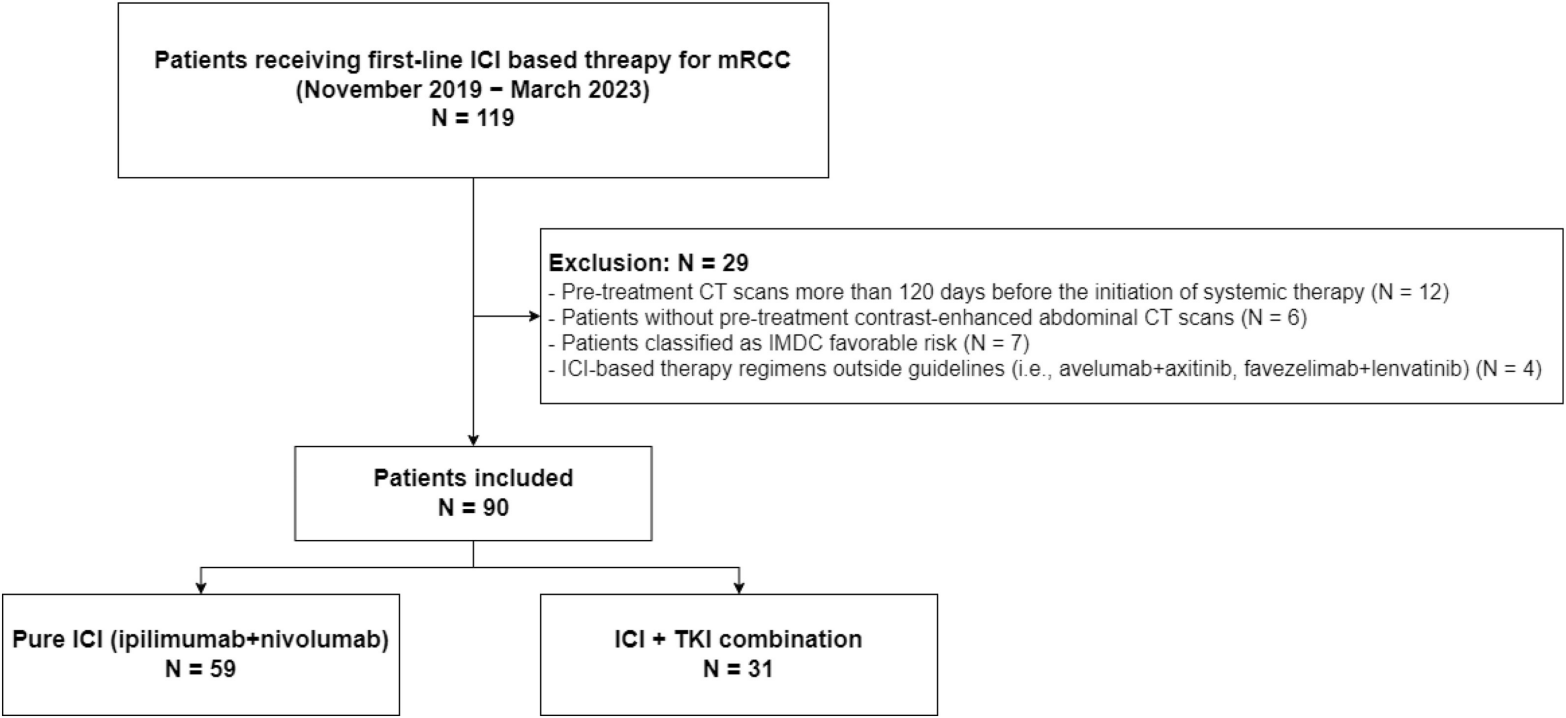
Patient selection process.

Patients with mRCC who received first‐line ICI‐based therapy at our institution between November 2019 and March 2023 were assessed. We specifically included patients classified as IMDC intermediate‐ and poor‐risk receiving therapy combinations acknowledged as first‐line treatments in the guidelines:PD‐1 inhibitor + CTLA‐4 inhibitor combination (ipilimumab + nivolumab) and PD‐1 inhibitor + TKI combinations (pembrolizumab + axitinib, pembrolizumab + lenvatinib or nivolumab + cabozantinib). The exclusion criteria included patients with pretreatment CT scans conducted more than 120 days before the initiation of systemic therapy, those without pretreatment contrast‐enhanced abdominal CT scans and those receiving ICI‐based therapy regimens outside the guidelines (e.g., avelumab + axitinib or favezelimab + lenvatinib) (Figure 1).

We reviewed the following variables: age; sex; body mass index (BMI); nephrectomy status (prior, upfront or deferred); time from diagnosis to systemic therapy; systemic therapy regimen; duration and number of ICI‐based therapy cycles; clinical nodal, visceral and bone metastasis status; histologic type; performance status; haemoglobin; corrected calcium; neutrophil count; and platelet count. Synchronous metastasis was defined as a diagnosis of metastasis within 3 months of the initial mRCC diagnosis. In contrast, metachronous metastasis was defined as a diagnosis of metastasis occurring more than 3 months after the initial diagnosis. BMI was calculated as follows: BMI (kg/m^2^) = ([weight]/[height]^2^). Patients were assigned a risk level according to the IMDC risk model [3].

Progression‐free survival (PFS) was defined as the duration from ICI‐based treatment initiation to radiographic disease progression or death from any cause, whichever occurred first. Overall survival (OS) was defined as the time from treatment initiation to the date of death or last follow‐up. Responses to systemic treatment were classified based on radiologic measurements using RECIST Version 1.1 [15], encompassing complete response, partial response, stable disease and progressive disease.

### 2.2 Image Analysis and Anthropometry

**FIGURE 2:**
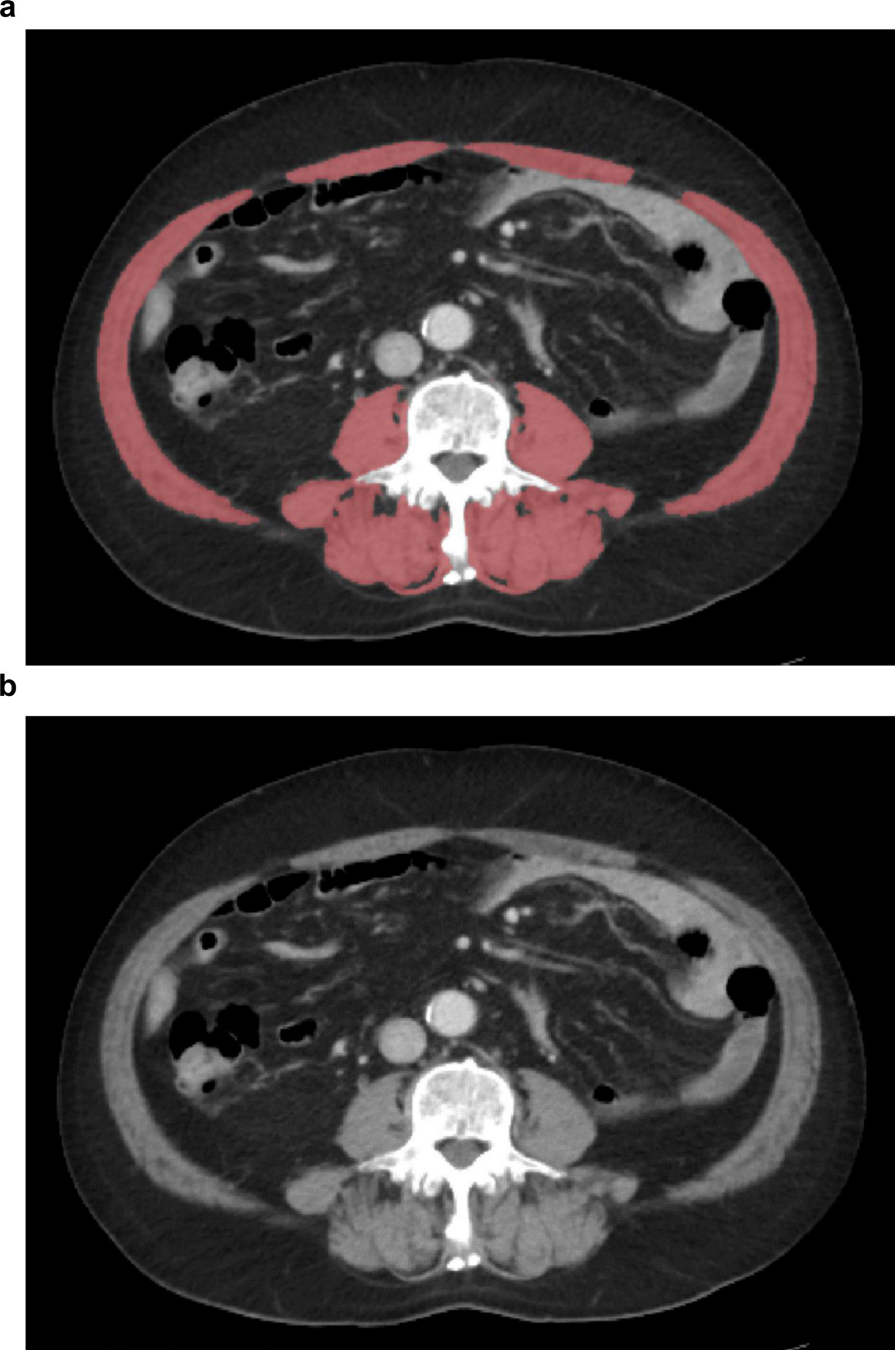
Automated segmentation of skeletal muscles. (a) Axial postcontrast computed tomography (CT) image at the third lumbar vertebra level in a 70‐year‐old male with a BMI of 28.9 kg/m^2^. (b) Visualization of segmented skeletal muscles highlighted in red overlays. SMI = 49.35 cm^2^/m^2^ and SMD = 24.21 HU, indicating myosteatosis in the patient. BMI, body mass index; HU, Hounsfield unit; SMI, skeletal muscle index; SMD, skeletal muscle density.

Pretreatment abdominal CT images (portal venous phase) were analysed using commercially available deep learning‐based software (DeepCatch v1.1.8; MedicalIP Co. Ltd., Seoul, Korea) (Figure 2). The third lumbar vertebra level was automatically selected, followed by segmentation and measurement of the cross‐sectional areas of skeletal muscles, including the rectus, transverse and oblique abdominal muscles, as well as psoas, and paraspinal muscles [S6]. A board‐certified radiologist (J.H.L.), with 8 years of experience in musculoskeletal imaging who was blinded to the patient details, confirmed the accuracy of the level selection and segmentation and ensured image quality (i.e., a streaking artefact from a metallic prosthesis). The cross‐sectional areas of the skeletal muscles in each patient (cm^2^) were normalized by dividing them by the square of the height (m^2^) to calculate the skeletal muscle index (SMI) [16]. Additionally, skeletal muscle density (SMD) (Hounsfield units [HU]) was determined by averaging the CT attenuation value of the voxels within these skeletal muscles.

To define LSMM, we applied cut‐off values of SMI of < 40.8 cm^2^/m^2^ for males and < 34.9 cm^2^/m^2^ for females, which are generally used among patients with cancer in Asian countries [17]. Myosteatosis was defined using the SMD with cut‐off values of < 41 HU for patients with BMI < 25 kg/m^2^ and < 33 HU for those with BMI ≥ 25 kg/m^2^ [18].

### 2.3 Single‐Cell mRNA Library Preparation and Sequencing

Single‐cell RNA sequencing was performed on samples from 12 patients selected from our main study cohort (7 patients without myosteatosis and 5 patients with myosteatosis). These patients were all diagnosed with clear cell RCC and were treatment‐naïve at the time of sample collection. Tissue samples were obtained prior to the initiation of systemic therapy. Following biopsy or surgical procedures, tissue and peripheral blood mononuclear cell (PBMC) samples were promptly transferred to the Samsung Genome Institute for sequencing within 1–3 h post‐collection. Each cell suspension underwent processing for scRNA‐seq library generation using either a Chromium Next GEM Single Cell 5' HT Reagent Kit v2 (Dual Index) or a Chromium Next GEM Single Cell 5' Reagent Kits v2 (Dual Index) (10× Genomics), strictly adhering to the manufacturer's protocol. Sequencing libraries were generated using Illumina sequencers (Nextseq_P3, NovaSeqX or MGI) according to the manufacturer's guidelines. The resulting reads were aligned to the GRCh38‐2020‐A human genome reference sequence and quantified using the CellRanger software (Version 5.0.1, 7.0.0).

### 2.4 scRNA‐Seq and Data Preprocessing

Using R v5.0.1 and Seurat package v5.0.1 [19], the unique molecular identifier count matrices of tissues and PBMCs from patients with mRCC were filtered using the following criteria: mitochondrial gene content < 25% and expressed gene number > 300. After normalizing the count matrices and analysing 2000 variable features, the 2000 most conserved genes with the highest median rank were selected using the SelectIntegrationFeatures function for data integration. Normalized data were converted to a natural log scale and projected through principal component analysis (PCA); integration anchors for the dataset were calculated using the FindIntegrationAnchors function. After identifying the mutual nearest neighbours using the IntegrateData function, the data were integrated using the anchor set. PCA projection was performed by converting the integrated data matrix to a natural log scale. Subsequently, uniform manifold approximation and projection (UMAP) dimension reduction was performed, and the FindNeighbor and FindCluster functions within the Louvain algorithm were used to detect clusters. Clusters with multiple canonical cell type markers [20] were considered doublets and were removed. Dimension reduction was performed again for the cell population (92 457 cells), from which the doublets were removed by finding variable features. PCA and batch effect correction were performed using the harmony package [21], and a UMAP map was created from the first 10 PCs.

### 2.5 Cell Type Identification and Sub‐cluster Analysis

The Louvain algorithm, implemented using the FindNeighbor and FindCluster functions, was employed to identify numerous clusters. Cell types were determined based on the expression of canonical cell type markers and differentially expressed genes (DEGs), as defined using the FindAllMarker function. Sub‐clusters of CD4^+^ T cells, CD8^+^ T cells, and myeloid cells were calculated for each cell type subset using the FindVariableFeatures, ScaleData, RunPCA, RunUMAP, FindNeighbor and FindCluster functions following the methodology described above. The CD4^+^ T [22], CD8^+^ T [23] and myeloid [24] cell subsets were further characterized using known cell type markers and DEGs.

### 2.6 Data Visualization and Statistical Analysis

We categorized the patients into two groups: those receiving a PD‐1 inhibitor + CTLA‐4 inhibitor combination and those receiving a PD‐1 inhibitor + TKI combination. Continuous variables are presented as the median and interquartile range (IQR), and the Mann–Whitney *U* test was used for comparison. For categorical variables, absolute counts (percentages) were reported, and comparisons were performed using Pearson's chi‐squared or Fisher's exact test. To examine the impact of LSMM and myosteatosis on treatment outcomes, Kaplan–Meier curves were generated, and the log‐rank test was used to compare PFS and OS between individuals with and without LSMM or myosteatosis within the PD‐1 inhibitor + CTLA‐4 inhibitor and PD‐1 inhibitor + TKI groups. A multivariate model was fitted using backward elimination to identify variables that significantly predicted survival after ICI treatment in patients with mRCC. The model was constructed using a stepwise approach, in which variables were sequentially removed based on their significance until the final set of predictors was obtained. Statistical analyses were performed using SPSS (Version 29.0; IBM, Armonk, NY, USA), with statistical significance set at *p* < 0.05.

All plotting and statistical analyses of single‐cell RNA‐seq data were performed using R 4.3.2. The Wilcoxon rank‐sum test was performed to compare the cell proportion and gene expression between Group 1 (patients without myosteatosis) and Group 2 (patients with myosteatosis). Statistical significance was set at *p* < 0.05. A gene set enrichment analysis (GSEA) was performed using the Reactome database, and Gene Ontology (GO) biological processes for the DEGs between Group 1 and Group 2 in CD8^+^ effector memory cells and non‐classical monocytes were identified using the msigdbr and fgsea packages. All plots were drawn using the Seurat and ggplot2 packages.

## 3 Results

### 3.1 Patient Characteristics

Ninety patients were retrospectively analysed in this study, with 59 in the PD‐1 inhibitor + CTLA‐4 inhibitor group and 31 in the PD‐1 inhibitor + TKI group (Figure 2). Table 1 provides demographic information and baseline disease characteristics. Most patients were male (73.3%), with a small majority (58.9%) younger than 65 years. Most patients had synchronous metastases (77.8%), and approximately half were classified as intermediate risk by IMDC criteria (51.1%). The median time interval between the pretreatment CT scan and initiation of systemic therapy was 32 days (IQR 18–48 days).

**TABLE 1:** Demographic data of patients treated with PD‐1 inhibitor + CTLA‐4 inhibitor or PD‐1 inhibitor + TKI regimens.

Regimen	PD‐1 inhibitor + CTLA‐4 inhibitor	PD‐1 inhibitor + TKI
*n*	59	31
Regimen, *n* (%)		
Ipilimumab + nivolumab	59 (100.0)	0 (0.0)
Nivolumab + cabozantinib	0 (0.0)	5 (16.1)
Pembrolizumab + axitinib	0 (0.0)	22 (71.0)
Pembrolizumab + lenvatinib	0 (0.0)	4 (12.9)
Sex, *n* (%)		
Female	19 (32.2)	5 (16.1)
Male	40 (67.8)	26 (83.9)
Age at treatment, median (IQR)	64 (56.8, 69.2)	55 (44, 65)
Age (65 cut‐off), *n* (%)		
< 65	31 (52.5)	22 (71.0)
≥ 65	28 (47.5)	9 (29.0)
Treatment cycle, median (IQR)	12 (6, 23)	7 (4, 12)
Treatment duration (month), median (IQR)	8.4 (4.0, 14.1)	4.2 (2.2, 9.1)
Type of metastasis, *n* (%)		
Synchronous (≤ 3 months)	46 (78.0)	24 (77.4)
Metachronous (> 3 months)	13 (22.0)	7 (22.6)
Nephrectomy, *n* (%)	33 (55.9)	19 (61.3)
BMI, median (IQR)	22.7 (21.0, 25.2)	23.0 (19.2, 26.5)
BMI (25 cut‐off), *n* (%)		
< 25	44 (74.6)	22 (71.0)
≥ 25	15 (25.4)	9 (29.0)
SMI, median (IQR)	46.1 (39.2, 51.2)	46.5 (40.7, 56.1)
LSMM, *n* (%)	11 (18.6)	6 (19.4)
SMD, median (IQR)	40.8 (32.9, 45.9)	43.8 (36.4, 51.3)
Myosteatosis, *n* (%)	28 (47.5)	9 (29.0)
Karnofsky Performance Status < 80%, *n* (%)	12 (20.3)	3 (9.7)
Time from diagnosis to systemic treatment < 1 year, *n* (%)	51 (86.4)	26 (83.9)
Haemoglobin < 13.6 ng/dL, *n* (%)	51 (86.4)	28 (90.3)
Neutrophils > 8.30 × 10^9^/L, *n* (%)	8 (13.6)	3 (9.7)
Platelets > 316 cells/μL, *n* (%)	26 (44.1)	8 (25.8)
Corrected calcium > 10.0 mg/dL, *n* (%)	11 (18.6)	2 (6.5)
IMDC risk, *n* (%)		
Intermediate	27 (45.8)	19 (61.3)
Poor	32 (54.2)	12 (38.7)
Clinical T stage, *n* (%)		
cT1	6 (10.2)	3 (9.7)
cT2	9 (15.3)	4 (12.9)
cT3	36 (61.0)	20 (64.5)
cT4	8 (13.6)	4 (12.9)
Clinical N stage, *n* (%)		
cN0	41 (69.5)	18 (58.1)
cN1	18 (30.5)	13 (41.9)
Fuhrmann grade, *n* (%)		
II	0 (0.0)	1 (6.2)
III	17 (58.6)	10 (62.5)
IV	12 (41.4)	5 (31.2)
Histology, *n* (%)		
Clear cell type	57 (96.6)	19 (61.3)
Non‐clear cell type	2 (3.4)	12 (38.7)
Papillary type	2	12
MiT family translocation	0	2
Unclassified	0	2
Sarcomatoid feature, *n* (%)	9 (17.3)	2 (7.1)
No. of metastasis, *n* (%)		
1	11 (18.6%)	5 (16.1%)
2	23 (39.0%)	11 (35.5%)
≥ 3 sites	25 (42.4%)	15 (48.4%)
Lung metastasis, *n* (%)	43 (72.9)	18 (58.1)
Liver metastasis, *n* (%)	11 (18.6)	8 (25.8)
Lymph node metastasis, *n* (%)	25 (42.4)	20 (64.5)
Bone metastasis, *n* (%)	18 (30.5)	13 (41.9)
Brain metastasis, *n* (%)	5 (8.5)	1 (3.2)

Abbreviations: BMI, body mass index; IMDC, International Metastatic Renal Cell Carcinoma Database Consortium; LSMM, low skeletal muscle mass; RCC, renal cell carcinoma; SMD, skeletal muscle density; SMI, skeletal muscle index.

Most patients in the PD‐1 inhibitor + TKI group were treated with pembrolizumab and axitinib (71%). The treatment patterns differed significantly between groups: PD‐1 inhibitor + CTLA‐4 inhibitor patients received more cycles (12 vs. 7 cycles) and longer therapy duration (median 8.4 vs. 4.2 months) compared to PD‐1 inhibitor + TKI patients. However, the median follow‐up period was similar, at 16.8 months (IQR 10.9–25.6 months) and 15.2 months (IQR 8.9–29.2 months), respectively (*p* = 0.902). The histological types differed between the groups, with 96.6% and 61.3% of patients in the PD‐1 inhibitor + CTLA‐4 inhibitor and PD‐1 inhibitor + TKI groups, respectively, having a clear cell type. Among non‐clear cell types, papillary RCC was the predominant subtype (71.4%). There were no significant differences in the number of metastases, with lung metastasis being the most common type.

The proportions of patients with LSMM and myosteatosis, as well as SMI and SMD, were comparable between the two groups. When comparing treatment responsiveness based on LSMM or myosteatosis within each group, no significant differences in the best response for these two muscle‐related parameters were observed in either group (Table S1).

During follow‐up, 29 patients (32.2%) died: 16 in the PD‐1 inhibitor + CTLA‐4 inhibitor group and 13 in the PD‐1 inhibitor + TKI group. Additionally, 51 patients (56.7%) experienced disease progression: 31 in the PD‐1 inhibitor + CTLA‐4 inhibitor group and 20 in the PD‐1 inhibitor + TKI group. For the overall cohort, the 1‐year mortality rate was 22.2% (95% CI, 14.9%–31.8%), and the 3‐month mortality rate was 6.7% (95% CI, 3.1%–13.8%). The 1‐year PFS rate was 53.3% (95% CI, 43.4%–65.4%), whereas the 3‐month PFS rate was 85.3% (95% CI, 78.3%–93.0%).

**FIGURE 3:**
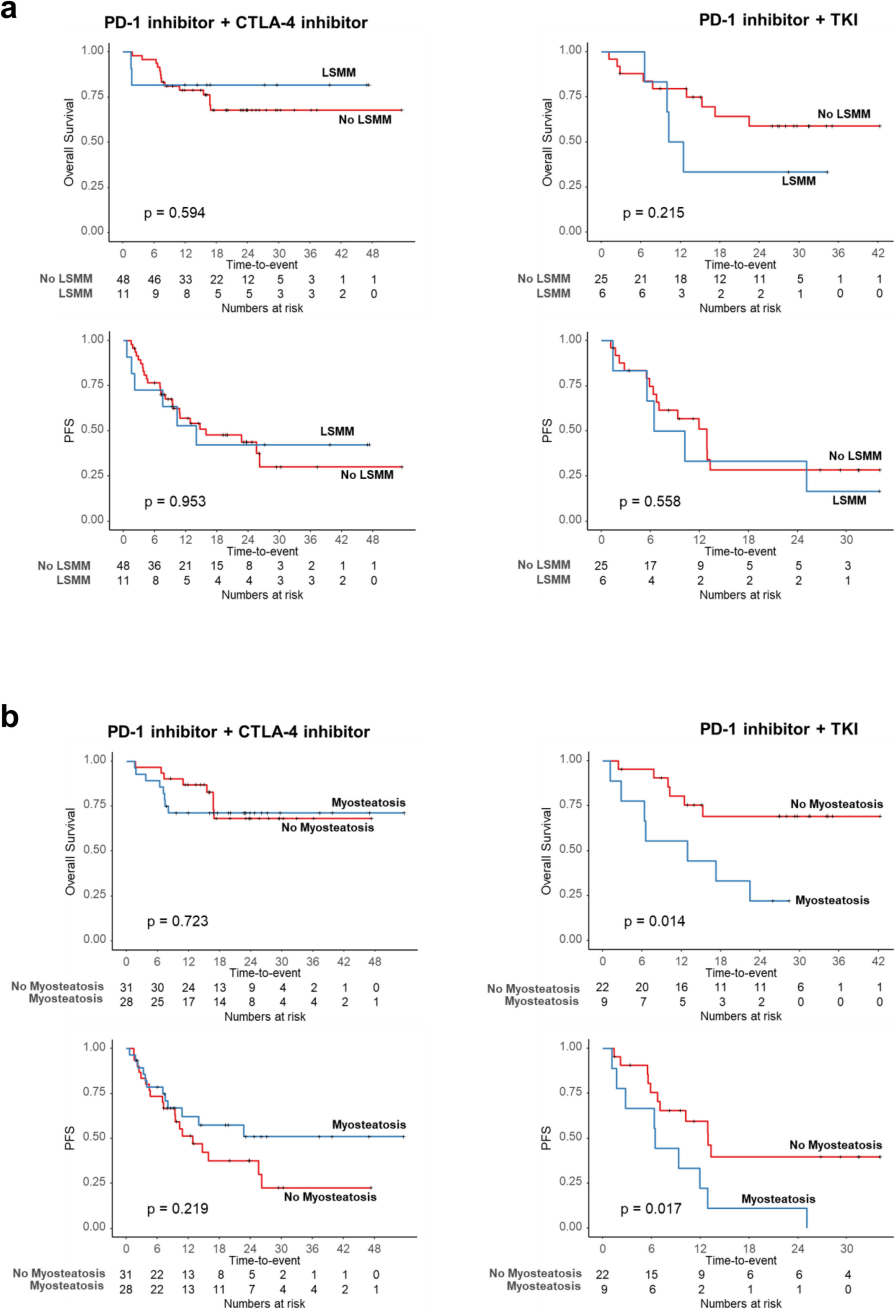
Kaplan–Meier curve analysis of overall survival (OS) and progression‐free survival (PFS) based on treatment regimen and muscle‐related parameters (low skeletal muscle mass [LSMM] and myosteatosis). (a) Kaplan–Meier curve analysis of OS and PFS according to treatment regimen and the presence of LSMM. (b) Kaplan–Meier curve analysis of OS and PFS according to treatment regimen and presence of myosteatosis.

Kaplan–Meier curve analysis revealed no significant differences in OS or PFS between LSMM and non‐LSMM patients in either group (all *p* > 0.05; Figure 3a). In contrast, myosteatosis had varying effects on survival (Figure 3b). Patients with myosteatosis in the PD‐1 inhibitor + CTLA‐4 inhibitor group had no difference in OS (*p* = 0.723) from those without and showed a trend towards improved PFS (*p* = 0.219). In contrast, those in the PD‐1 inhibitor + TKI group exhibited lower OS (*p* = 0.014) and worse PFS than patients without myosteatosis (*p* = 0.017). Multivariable analysis confirmed myosteatosis as an independent prognostic factor for poorer OS in the PD‐1 inhibitor + TKI group (hazard ratio [HR], 5.39; 95% CI, 1.54–18.80; *p* = 0.008) and improved PFS in the PD‐1 inhibitor + CTLA‐4 inhibitor group (HR, 0.46; 95% CI, 0.21–0.99; *p* = 0.049), along with factors such as poor IMDC risk status, nephrectomy, positive nodal status and multiple metastatic sites. LSMM was not associated with OS in either treatment group (Tables 2 and 3).

**TABLE 2:** Cox regression analysis of overall survival.

A. PD‐1 inhibitor + CTLA‐4 inhibitor				
	Crude HR (95% CI)	Crude *p*	Adj. HR (95% CI)	Adj. *p*
Sex: Male vs. female	0.72 (0.26, 1.99)	0.529		
Type of metastasis: Metachronous vs. synchronous	0.20 (0.03, 1.54)	0.123		
Any nephrectomy	0.37 (0.14, 1.04)	0.058	0.54 (0.19, 1.51)	0.238
Age: ≥ 65 vs. < 65	1.27 (0.48, 3.4)	0.628		
BMI: ≥ 25 vs. < 25	0.65 (0.19, 2.29)	0.506		
Myosteatosis: Yes vs. no	1.19 (0.45, 3.17)	0.730		
LSMM: Yes vs. no	0.67 (0.15, 2.94)	0.595		
IMDC risk: Poor vs. intermediate	2.86 (0.92, 8.86)	0.069	3.00 (0.96, 9.35)	0.059
cT stage: III/IV vs. I/II	1.00 (0.32, 3.09)	0.993		
Clinical nodal status: Positive vs. negative	1.09 (0.38, 3.13)	0.879		
No. of metastasis: ≥ 3 site vs. 1–2 site	4.25 (1.45, 12.47)	0.008	3.96 (1.33, 11.81)	0.014

B. PD‐1 inhibitor + TKI				
	Crude HR (95% CI)	Crude *p*	Adj. HR (95% CI)	Adj. *p*
Sex: Male vs. female	2.70 (0.35, 20.76)	0.341		
Type of metastasis: Metachronous vs. synchronous	0.25 (0.03, 1.92)	0.183		
Any nephrectomy	0.22 (0.07, 0.68)	0.009	0.33 (0.08, 1.42)	0.137
Age: ≥ 65 vs. < 65	1.80 (0.59, 5.51)	0.303		
BMI: ≥ 25 vs. < 25	0.86 (0.24, 3.14)	0.822		
Myosteatosis: Yes vs. no	3.59 (1.2, 10.72)	0.022	5.39 (1.54, 18.80)	0.008
LSMM: Yes vs. no	2.09 (0.64, 6.84)	0.225		
IMDC risk: Poor vs. intermediate	6.28 (1.91, 20.63)	0.002	6.51 (1.71, 24.69)	0.006
cT stage: III/IV vs. I/II	0.94 (0.26, 3.42)	0.924		
Clinical nodal status: Positive vs. negative	1.85 (0.62, 5.54)	0.268		
No. of metastasis: ≥ 3 site vs. 1–2 site	2.70 (0.85, 8.51)	0.091	3.37 (0.69, 16.55)	0.135
Histology: Non‐clear cell vs. clear cell	1.94 (0.64, 5.82)	0.240	0.86 (0.18, 4.25)	0.857

**TABLE 3:** Cox regression analysis of progression‐free survival.

A. PD‐1 inhibitor + CTLA‐4 inhibitor				
	Crude HR (95% CI)	Crude *p*	Adj. HR (95% CI)	Adj. *p*
Sex: Male vs. female	0.70 (0.33, 1.45)	0.335	0.51 (0.23, 1.14)	0.103
Type of metastasis: Metachronous vs. synchronous	1.03 (0.46, 2.32)	0.946		
Any nephrectomy	0.54 (0.26, 1.09)	0.086	0.32 (0.14, 0.78)	0.012
Age: ≥ 65 vs. < 65	0.92 (0.45, 1.88)	0.827		
BMI: ≥ 25 vs. < 25	0.92 (0.39, 2.13)	0.838		
Myosteatosis: Yes vs. no	0.64 (0.31, 1.31)	0.223	0.46 (0.21, 0.99)	0.049
LSMM: Yes vs. no	0.97 (0.4, 2.4)	0.953		
IMDC risk: Poor vs. intermediate	1.74 (0.84, 3.59)	0.137	1.56 (0.70, 3.48)	0.275
cT stage: III/IV vs. I/II	0.98 (0.42, 2.29)	0.969		
Clinical nodal status: Positive vs. negative	0.58 (0.25, 1.35)	0.203	0.32 (0.13, 0.83)	0.018
No. of metastasis: ≥ 3site vs. 1–2 site	1.70 (0.82, 3.53)	0.151		

B. PD‐1 inhibitor + TKI				
	Crude HR (95% CI)	Crude *p*	Adj. HR (95% CI)	Adj. *p*
Sex: Male vs. female	2.68 (0.62, 11.62)	0.189		
Type of metastasis: Metachronous vs. synchronous	0.99 (0.36, 2.74)	0.991		
Any nephrectomy	0.51 (0.21, 1.25)	0.142		
Age: ≥ 65 vs. < 65	2.19 (0.86, 5.61)	0.102		
BMI: ≥ 25 vs. < 25	1.17 (0.45, 3.07)	0.743		
Myosteatosis: Yes vs. no	2.85 (1.16, 6.96)	0.022	2.93 (1.17, 7.33)	0.022
LSMM: Yes vs. no	1.34 (0.49, 3.71)	0.568		
IMDC risk: Poor vs. intermediate	2.42 (1, 5.85)	0.050	3.87 (1.36, 10.99)	0.011
cT stage: III/IV vs. I/II	0.96 (0.35, 2.65)	0.936		
Clinical nodal status: Positive vs. negative	1.50 (0.61, 3.64)	0.375		
No. of metastasis: ≥ 3site vs. 1–2 site	2.32 (0.92, 5.8)	0.073	3.03 (1.11, 8.25)	0.030
Histology: Non‐clear cell vs. clear cell	0.70 (0.27, 1.84)	0.473	0.44 (0.14, 1.36)	0.152

Sensitivity analyses of the clear cell RCC subgroup (*n* = 76) showed similar directional trends, although statistical significance was not reached. Myosteatosis was associated with poorer outcomes in the PD‐1 inhibitor + TKI group (OS: HR, 3.96; 95% CI, 2.85–5.51; *p* = 0.169; PFS:HR, 2.88; 95% CI, 1.63–5.09; *p* = 0.290) and improved PFS in the PD‐1 inhibitor + CTLA‐4 inhibitor group (HR, 0.36; 95% CI, 0.20–0.66; *p* = 0.306), compared with those in patients without myosteatosis (Table S3 and Supplementary Figure S4).

### 3.2 Cell Type Identification Reveals Differences in Tissue Proportions Between the two Groups

**FIGURE 4:**
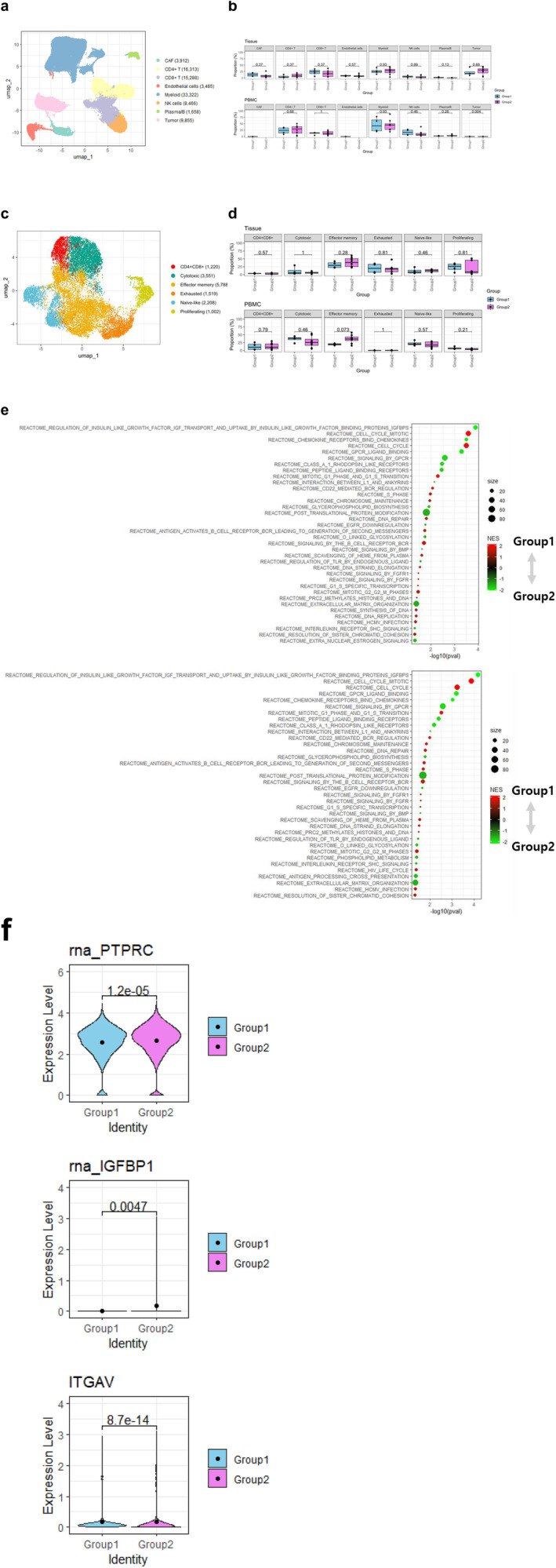
Single‐cell RNA‐seq analysis and differences in CD8^+^ T cells: Group 1 (without myosteatosis, *n* = 7) vs. Group 2 (with myosteatosis, *n* = 5). (a) UMAP plot of 92 288 cells from 12 patients with mRCC, coloured by global cell types. (b) Proportions of global cell subtypes in each sample according to myosteatosis group. (c) UMAP plot of CD8^+^ T cells, coloured by cell subtypes. (d) Proportions of CD8^+^ T‐cell subtypes in each sample according to myosteatosis group. (e) GSEA using the Reactome database for DEGs between Group 1 and Group 2 in CD8^+^ effector memory cells; (above) GSEA results for tissue; (below) GSEA results for PBMCs. DEGs *p*.adj < 0.05, GSEA *p* < 0.05. (f) Differential gene expression related to immunotherapy response between two groups in all CD8^+^ effector memory cells. Dots represent the mean expression.

To explore the mechanistic differences in how myosteatosis affects survival outcomes between the PD‐1 inhibitor + CTLA‐4 inhibitor and PD‐1 inhibitor + TKI groups, we conducted scRNA‐seq. We aimed to determine the immune cell characteristics associated with treatment response by analysing scRNA‐seq data from 92 457 cells, derived from both tissue samples and PBMCs of 12 patients with mRCC at the Samsung Genome Institute. Patient characteristics for this subcohort are provided in Table S2. Based on canonical marker gene expression, eight cell types were identified through unbiased clustering: tumour cells, endothelial cells, cancer‐associated fibroblasts (CAFs), CD4^+^ T, CD8^+^ T, natural killer, plasma/B and myeloid cells (Figure 4a and Figure S1a,b). To confirm the proportional differences in cell types between the groups at each sample type, the cells were categorized into tissues and PBMCs. In tissue samples, CAFs and CD8^+^ T cells were more abundant in patients without myosteatosis, whereas CD4^+^ T cells were more abundant in patients with myosteatosis (Figure 4b). In PBMCs, CD4^+^ T, CD8^+^ T, natural killer and myeloid cells represented the majority of cells; however, the individual proportions did not differ markedly between the groups.

### 3.3 Patients With Myosteatosis Have a High Proportion of CD8^+^ Effector Memory Cells and Elevated IGFBP1 Expression

We investigated whether there were distinct features of CD8^+^ T cell sub‐clusters between patient groups with and without myosteatosis. Among the 15 288 cells comprising the CD8^+^ T sub‐clusters, we identified six subtypes: CD4^+^CD8^+^, cytotoxic, effector memory, exhausted, naïve and proliferating (MKI67^+^) (Figure 4c and Figure S2a–c). Effector memory cells were more abundant in patients with myosteatosis than in those without myosteatosis, with a relatively pronounced difference observed in PBMCs (*p* = 0.073; Figure 4d). Using Reactome database analysis, we found that the term ‘REGULATION OF INSULIN‐LIKE GROWTH FACTOR (IGF) TRANSPORT AND UPTAKE BY IGF BINDING PROTEINS (IGFBPs)’ was most significantly associated with effector memory cells in both tissues and PBMCs (Figure 4e). TEMRA, identified by CD45RA^+^CCR7^−^ expression in effector memory cells, was associated with a higher proportion of responders in pretreatment samples for PD‐1 inhibitor + CTLA‐4 inhibitor combination therapy [25]. The *PTPRC* gene encoding CD45RA was significantly upregulated in the effector memory cells of patients with myosteatosis (Figure 4f). The expression of *IGFBP1* and *ITGAV—*associated with TKI resistance [26]—was also significantly upregulated in these cells, providing evidence of the protective effects of anti‐PD‐1 + CTLA‐4 inhibitor therapy and resistance to anti‐PD‐1 + TKI therapy in patients with myosteatosis.

### 3.4 Immune Checkpoint Molecules Are Upregulated in CD4^+^ T, CD8^+^ T and Treg Cells in Patients With Myosteatosis

**FIGURE 5:**
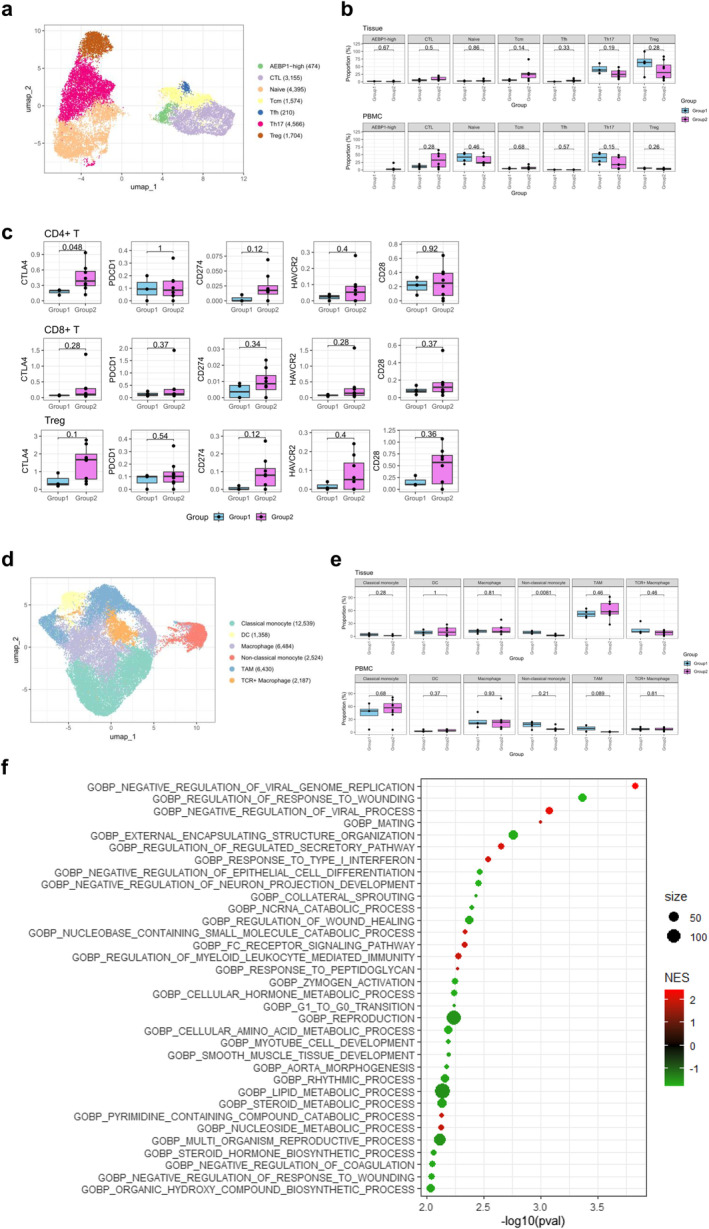
Differential expression of immune checkpoint molecules in T cells and monocytes: Group 1 (without myosteatosis, *n* = 7) vs. Group 2 (with myosteatosis, *n* = 5). (a) UMAP plot of CD4^+^ T cells, coloured by cell subtypes. (b) Proportions of CD4^+^ T‐cell subtypes in each sample according to myosteatosis group. (c) Higher expression of immune checkpoint molecules in CD4^+^ T, CD8^+^ T, and Tregs of the tumour microenvironment in a group of patients with myosteatosis. A patient with a singular CD4^+^ T‐cell count was excluded from the CD4^+^ T and Treg plot. (d) UMAP plot of myeloid cells, coloured by cell subtypes. (e) Proportions of myeloid cell subtypes in each sample according to myosteatosis group. (f) GSEA using GO biological process for DEGs of non‐classical monocytes. DEGs *p*.adj < 0.05, GSEA *p* < 0.01.

Given that all patients received combination therapies based on PD‐1 inhibitor treatment, we investigated the expression of immune checkpoint molecules (ICMs) in T cells. Sub‐clustering of the CD4^+^ T‐cell subset (16 313 cells) from the global cell population revealed seven distinct subtypes identified by canonical marker gene expression: *AEBP1*‐high, naïve, cytotoxic T lymphocytes (CTLs), central memory T cells (Tcm), follicular helper T cells (Tfh), Th17 and T regulatory (Treg) cells (Figure 5a and Figure S3a,b). When comparing the proportions of CD4^+^ T‐cell subtypes in tissues and PBMCs, CTLs, Tcm, Th17 and Tregs constituted the tissue cell population (Figure 5b). Tcm cells were more prevalent in patients with myosteatosis, whereas Th17 and Treg cells were more abundant in patients without myosteatosis. In PBMCs, naïve cells, CTLs and Th17 cells predominated, with naïve and Th17 cells slightly more prevalent in patients without myosteatosis and CTLs more prevalent in patients with myosteatosis.

To elucidate the association between immunotherapy combinations and responses across groups, we investigated the expression of five major ICMs—CTLA4, PDCD1, CD274, HAVCR2 and CD28—in CD4^+^ and CD8^+^ T cells from tissues (Figure 5c) [27]. In CD4^+^ T cells, CTLA4 (*p* = 0.048), CD274 and HAVCR2 were highly expressed in patients with myosteatosis. In CD8^+^ T cells, all ICMs were highly expressed in patients with myosteatosis. CTLA4, the target of CTLA‐4 inhibitors such as ipilimumab, is primarily expressed in Tregs under the control of FOXP3 [28]. Further, when comparing the expression of each ICM in Tregs by group, all except PDCD1 (i.e., CTLA4, CD274, HAVCR2 and CD28) were highly expressed in patients with myosteatosis, mirroring the trend observed in the whole‐cell population of CD4^+^ T and CD8^+^ T cells (Figure 5c).

### 3.5 An Increased Proportion of Anti‐tumoural Non‐classical Monocytes Is Present in Patients Without Myosteatosis

After sub‐clustering myeloid cells (31 522 cells) from both tissues and PBMCs, we identified six distinct subtypes: classical monocytes, non‐classical monocytes, macrophages, TCR + macrophages, tumour‐associated macrophages and dendritic cells (Figure 5d and Figure 3c–d). Notably, non‐classical monocytes were significantly more abundant in tissue samples from patients without myosteatosis than in those from patients with myosteatosis (Figure 5e). The DEGs in non‐classical monocytes were analysed using GSEA with GO biological process gene sets (Figure 5f). Our findings revealed the upregulation of terms related to ‘NEGATIVE REGULATION OF VIRAL GENOME REPLICATION’ and ‘RESPONSE TO TYPE 1 INTERFERON’, whereas terms associated with ‘REGULATION OF RESPONSE TO WOUNDING’ and ‘REGULATION OF WOUND HEALING’ were downregulated (*p* < 0.01).

## 4 Discussion

To our knowledge, this is the first study to investigate the prognostic impact of muscle‐related parameters on treatment outcomes in patients with mRCC undergoing first‐line ICI‐based therapy. We conducted separate analyses for PD‐1 inhibitor + CTLA‐4 inhibitor and PD‐1 inhibitor + TKI combinations, along with providing biological evidence using scRNA‐seq. Our analysis revealed distinct prognostic implications for myosteatosis in the two treatment groups.

In the PD‐1 inhibitor + TKI group, patients with myosteatosis exhibited significantly poorer OS and PFS than those without myosteatosis. This finding is consistent with a previous study on ICI‐based therapy in mRCC, which reported that decreased SMD, linked to myosteatosis, is associated with unfavourable OS and PFS [6]. The detrimental impact of myosteatosis in this group may be attributed to the effects of TKIs on the muscle tissue. TKIs target enzymes that activate intracellular molecular pathways, including the PI3K–AKT–mTOR pathway, which is crucial for muscle protein synthesis and maintenance [29, 30]. The inhibition of this pathway by TKIs may exacerbate muscle deterioration in patients with pre‐existing myosteatosis, potentially intensifying treatment toxicities and worsening clinical outcomes [31, 32].

Our study revealed a significant protective effect of pretreatment myosteatosis on PFS in the PD‐1 + CTLA‐4 inhibitor group. This finding contrasts with previous research that reported poor outcomes associated with myosteatosis in patients treated with ICI‐based therapy, primarily with anti‐PD‐1 monotherapy [6]. Our results suggest that, in patients with myosteatosis, blocking the CTLA‐4 pathway may impact therapeutic efficacy more than blocking the PD‐1/PD‐L1 pathway.

scRNA‐seq provided insights into the potential mechanisms underlying these differential effects. We observed increased CD4^+^ T‐cell infiltration and notably higher CTLA‐4 expression in CD4^+^ T cells from patients with myosteatosis than from those without myosteatosis. Additionally, checkpoint molecules, particularly CTLA‐4, but not PD‐1, were elevated in Tregs from patients with myosteatosis. These findings further support the notion that the CTLA‐4 blockade may lead to better clinical outcomes in patients with myosteatosis.

The contrasting effects of PD‐1 and CTLA‐4 blockades on Treg function may explain the differences between the treatment groups. PD‐1 regulates Treg activity, and its blockade can enhance Treg activation, potentially contributing to cancer‐type progression [33, 34, 35]. This may partially explain the poor outcomes observed in the PD‐1 inhibitor + TKI group for patients with myosteatosis. Conversely, CTLA‐4 is crucial for Treg immunosuppressive function, and its inhibition likely reduces Treg activity [33, 34, 35]. This effect could be particularly beneficial for patients with myosteatosis, in whom we observed an increased expression of ICMs in Tregs.

Our analysis also revealed increased CD8^+^ effector memory cells in both tissues and PBMCs from patients with mRCC with myosteatosis. These cells exhibited elevated IGF transport and uptake by IGFBP, with significant IGFBP1 upregulation in patients with myosteatosis. Recent studies have shown that IGFBP1 enhances resistance to anti‐angiogenic TKIs in hepatocellular carcinoma [26]. Additionally, we observed increased expression of CD45RA, a marker of terminally differentiated effector memory cells, in patients with myosteatosis. TEMRA cells are reportedly important for combined anti‐PD‐1 and anti‐CTLA‐4 blockade treatment efficacy [25]. These findings suggest that the gene expression profile and cell type ratios of CD8^+^ effector memory cells may be important indicators of patient responsiveness to PD‐1 inhibitor + CTLA‐4 inhibitor and PD‐1 inhibitor + TKI treatment in patients with mRCC with myosteatosis.

Furthermore, we found a higher proportion of non‐classical monocytes in patients without myosteatosis than in those with myosteatosis. These cells are typically beneficial in anti‐inflammatory responses, vascular repair and organ recovery during kidney injury [36]. Coincidently, the increased presence of non‐classical monocytes, along with their enhanced Type 1 interferon response, which inhibits angiogenesis and promotes an anti‐tumour immune response in the TME, may contribute to a better response to PD‐1 inhibitor + TKI treatment in patients without myosteatosis [37].

Our findings illuminate potential similarities between myosteatosis and obesity‐related immune responses in patients with mRCC with regard to CD8^+^ T‐cell dysfunction, a key feature of obesity‐induced chronic inflammation. Recent findings from a study on diet‐induced obesity in mice demonstrated an increase in exhausted CD8^+^ tumour‐infiltrating lymphocytes (TILs) characterized by markers such as PD‐1, TIM3 and LAG3, along with a reduction in proliferating CD8^+^ TILs marked by Ki67 expression [38]. However, in our single‐cell data, the frequency of exhausted (PDCD1^+^HAVCR2^+^LAG3^+^) or proliferating (MKI67^+^) CD8^+^ T cells was significantly variable among patients at the sub‐cluster level, and we did not observe consistent trends. Nevertheless, an upregulation of PD‐1, PD‐L1 (CD274) and TIM3 expression was observed in the CD8^+^ T cells from patients with myosteatosis (Group 2) compared to that in patients without myosteatosis (Group 1). Another recent multi‐omics study analysed immune cell phenotypes in obese mice [39]. This study reported a reduction in Tregs and an increase in CD8^+^ effector memory T cells in obesity, with a concomitant upregulation of exhaustion markers in CD8^+^ T cells. These findings are consistent with our results: increased effector memory T cells, decreased Tregs and increased expression of exhaustion‐associated molecules in CD8^+^ T cells from patients with myosteatosis. Taken together, these data suggest that myosteatosis, much like obesity, can create a chronic inflammatory microenvironment that promotes T‐cell dysfunction, including exhaustion. Furthermore, the chronic inflammatory state associated with myosteatosis likely influences both local and systemic immune responses, suggesting that muscle composition may serve as an important biomarker for immunotherapy response prediction. The condition is part of a broader ‘metabaging cycle’, where lipid metabolism dysfunction and chronic inflammation in fat and muscle tissues interact and propagate systemically [7]. The interplay between metabolic and inflammatory processes significantly impacts overall health and therapeutic responses, highlighting the potential of muscle composition as a predictive marker in immunotherapy.

Our study found that LSMM did not affect OS or PFS in either treatment group. This adds to the current conflicting evidence in the literature regarding the prognostic value of LSMM in patients with mRCC receiving ICI‐based therapy. Some have reported associations between a lower SMI and better PFS in patients treated with first‐line ipilimumab and nivolumab [5], whereas others have found no significant impact exerted by LSMM on prognostic predictions in patients receiving nivolumab monotherapy [40]. The observed heterogeneity in the prognostic significance of SMI/LSMM across studies can be attributed to several methodological and clinical factors, including the inherent variability in study populations, therapeutic protocols [5] and inconsistent application of SMI cut‐off values and LSMM definitions across research groups [16].

Our findings suggest that muscle composition assessment, particularly myosteatosis status, could serve as an additional factor in treatment decision‐making for patients with mRCC. The differential outcomes observed indicate that pretreatment CT evaluation of muscle composition might help guide optimal treatment selection. This assessment could be readily implemented in clinical practice, as CT imaging is routinely performed for staging, and muscle density measurement can be performed using widely available imaging software. However, myosteatosis status should be considered alongside other clinical factors, such as IMDC risk criteria and PD‐L1 status, rather than in isolation. The integration of muscle composition assessment into clinical decision‐making will require validation through larger, prospective studies to confirm its predictive value across different patient populations. Additionally, given the exploratory nature of our analyses, these findings should be considered hypothesis‐generating, and the association between muscle composition and treatment outcomes needs clear validation in large cohorts.

Our study has several limitations. First, the retrospective, single‐centre design may introduce biases and limit the generalizability of our findings to other institutions with different patient populations or clinical practices. Consistency in treatment protocols and data collection methods, however, has ensured greater control over study variables. Second, the relatively small sample size in certain subsets resulted in statistical power below the conventional threshold of 80% for some analyses. Post hoc power analysis revealed powers of 69.6% for PFS in the PD‐1 + CTLA‐4 inhibitor subgroup, 78.4% for PFS in the TKI subgroup and 93.3% for OS in the PD‐1 + TKI subgroup. Despite these limitations, mechanistic insights from experimental data support the robustness of our findings, and larger prospective studies are needed to validate these results. Third, including patients with non‐clear cell RCC alongside clear cell RCC cases may raise concerns about histological heterogeneity. This concern is mitigated by the predominance of papillary RCC cases among non‐clear cell subtypes, which demonstrate treatment responses similar to those of clear cell RCC in immunotherapy. Furthermore, histological type was not employed as an independent predictor in multivariate analysis, and consistent trends in sensitivity analyses were limited to patients with clear cell RCC. Additionally, scRNA‐seq focused exclusively on clear cell RCC samples, ensuring biological consistency. Finally, although our scRNA‐seq analysis provides valuable insights into the immune microenvironment, the pretreatment nature of our data and the small sample size warrant further research with larger cohorts at both pre‐ and post‐treatment levels to better capture molecular dynamics.

In conclusion, this study highlights the differential impact of myosteatosis on treatment outcomes in patients with mRCC receiving first‐line ICI‐based therapy. Myosteatosis negatively affected OS and PFS in patients treated with PD‐1 inhibitor + TKI while offering a protective effect on PFS in patients treated with PD‐1 inhibitor + CTLA‐4 inhibitor. These findings underscore the need for personalized treatment strategies based on muscle composition. Furthermore, scRNA‐seq analysis revealed important differences in T cells in the immune microenvironment between patients with and without myosteatosis, providing potential mechanistic insights into the observed treatment responses.

## Author Contributions

Minyong Kang and Ji Hyun Lee participated in the conception and design of the study. Jiwoong Yu, Hyeonju Ahn, Kyung Yeon Han, Woong‐Yang Park, and Minyong Kang carried out the analysis and interpretation of data and the writing of the manuscript. Wan Song, Hyun Hwan Sung, Hwang Gyun Jeon, Byong Chang Jeong, Seong Il Seo, Seong Soo Jeon, and Se Hoon Park were involved in the collection of tissues. Jiwoong Yu and Hyeonju Ahn were involved in the processing of scRNA‐seq data. Ji Hyun Lee participated in the study design and coordination and helped to draft the manuscript. All authors have read and approved the final version of the manuscript.

## Conflicts of Interest

The authors declare no conflicts of interest.

## Supporting information


**Table S1** Best responses to systemic treatment.
**Table S2.** Baseline clinical characteristics of patients with myosteatosis based on single‐cell RNA‐seq analysis.
**Table S3.** Univariate Cox analysis of myosteatosis for OS and PFS in two treatment groups of the clear cell RCC‐only cohort.

**Data S1** Supporting Information.

**Figure S1** Global cell type annotation. (a) UMAP plot of 92 457 cells from 12 patients with mRCC, coloured by 18 clusters. (b) Expression of marker genes for eight global cell types. Cluster 17 (169 cells) was removed because marker gene expression was not confirmed.

**Figure S2** CD8^+^ T‐cell sub‐clustering. (a) UMAP plot of CD8^+^ T cells from 12 patients with mRCC, coloured by nine clusters. (b) Dot plot showing marker gene expression in CD8^+^ T cells. (c) Heatmap showing the expression of the top five DEGs from each cluster in CD8^+^ T cells.

**Figure S3** Sub‐clustering of CD4^+^ T cells and myeloid cells. (a) UMAP plot of CD4^+^ T cells from 12 patients with mRCC, coloured by 14 clusters. (b) Expression of marker genes of CD4^+^ T subtypes. (c) UMAP plot of myeloid cells from 12 patients with mRCC, coloured by 10 clusters, sample type and myosteatosis group. (d) Expression of marker genes of myeloid cell subtypes.

**Figure S4** Kaplan–Meier curve analysis of overall survival (OS) and progression‐free survival (PFS) according to treatment regimen and presence of myosteatosis in the clear cell RCC‐only cohort.

## Data Availability

All data associated with this study are presented in the paper. Single‐cell RNA sequencing data supporting the result of this study will be provided by the corresponding author upon reasonable request. The data are not publicly available due to privacy and ethical constraints. The data are deposited in the controlled‐access data repository of Samsung Medical Center.
