# Supplementary material for: Paradoxical Effect of Myosteatosis on the Immune Checkpoint Inhibitor Response in Metastatic Renal Cell Carcinoma
Source: J Cachexia Sarcopenia Muscle. 2025 Mar 7;16(2):e13758. doi: 10.1002/jcsm.13758 (PMC11886412; doi:10.1002/jcsm.13758)
Supplement: Supplementary file 1 — Table S1 Best responses to systemic treatment. Table S2. Baseline clinical characteristics of patients with myosteatosis based on single‐cell RNA‐seq analysis. Table S3. Univariate Cox analysis of myosteatosis for OS and PFS in two treatment groups of the clear cell RCC‐only cohort. [file JCSM-16-e13758-s002.docx]

**Supplementary Table S1. Best responses to systemic treatment**

**A. PD-1 inhibitor + CTLA-4 inhibitor**

|  | No LSMM | LSMM | *p* | No Myosteatosis | Myosteatosis | *p* |
| --- | --- | --- | --- | --- | --- | --- |
| CR | 2 (4.2%) | 2 (18.2%) | 0.207 | 1 (3.2%) | 3 (10.7%) | 0.389 |
| PR | 27 (56.3%) | 3 (27.3%) |  | 14 (45.2%) | 16 (57.1%) |  |
| SD | 10 (20.8%) | 3 (27.3%) |  | 8 (25.8%) | 5 (17.9%) |  |
| PD | 9 (18.8%) | 3 (27.3%) |  | 8 (25.8%) | 4 (14.3%) |  |

|  | No LSMM | LSMM | *p* | No Myosteatosis | Myosteatosis | *p* |
| --- | --- | --- | --- | --- | --- | --- |
| ORR | 29 (60.4%) | 5 (45.5%) | 0.502 | 15 (48.4%) | 19 (67.9%) | 0.188 |

LSMM, Low skeletal muscle mass; ORR, Objective response rate

**B. PD-1 inhibitor + TKI**

|  | No LSMM | LSMM | *p* | No Myosteatosis | Myosteatosis | *p* |
| --- | --- | --- | --- | --- | --- | --- |
| CR | 1 (4.0%) | 2 (33.3%) | 0.132 | 2 (9.1%) | 1 (11.1%) | 0.292 |
| PR | 13 (52.0%) | 3 (50.0%) |  | 13 (59.1%) | 3 (33.3%) |  |
| SD | 5 (20.0%) | 0 (0%) |  | 4 (18.2%) | 1 (11.1%) |  |
| PD | 6 (24.0%) | 1 (16.7%) |  | 3 (13.6%) | 4 (44.4%) |  |

|  | No LSMM | LSMM | *p* | No Myosteatosis | Myosteatosis | *p* |
| --- | --- | --- | --- | --- | --- | --- |
| ORR | 14 (56.0%) | 5 (83.3%) | 0.363 | 15 (68.2%) | 4 (44.4%) | 0.253 |

LSMM, Low skeletal muscle mass; ORR, Objective response rate

**Supplementary Table S2.** **Baseline clinical characteristics of patients with myosteatosis based on single-cell RNA-seq analysis**

|  | Group 1^†^ | Group 2^‡^ |
| --- | --- | --- |
| *n* | 4 | 8 |
| IO regimens (%) |  |  |
| Ipilimumab + Nivolumab | 4 (100.0) | 8 (100.0) |
| Age (65 cut off) (%) |  |  |
| < 65 | 4 (100.0) | 3 (37.5) |
| ≥ 65 | 0 | 5 (62.5) |
| Sex (%) |  |  |
| Male | 3 (75.0) | 6 (75.0) |
| Female | 1 (25.0) | 2 (25.0) |
| Histologic subtype (%) |  |  |
| Clear cell | 4 (100.0) | 8 (100.0) |
| Papillary | 0 (0.0) | 0 (0.0) |
| IMDC (Heng) risk group (%) |  |  |
| Favorable | 0 (0.0) | 0 (0.0) |
| Intermediate | 3 (75.0) | 6 (75.0) |
| Poor | 1 (25.0) | 2 (25.0) |
| No. of metastasis (%) |  |  |
| 1 | 3 (75.0) | 5 (62.5) |
| 2 | 0 (0.0) | 2 (25.0) |
| ≥ 3 sites | 1 (25.0) | 1 (12.5) |

^†^Group of patients with mRCC without myosteatosis

^‡^Group of patients with mRCC with myosteatosis

**Supplementary Table S3. Univariate Cox analysis of myosteatosis for OS and PFS in two treatment groups of the clear cell RCC-only cohort**

| **PD-1 inhibitor + CTLA-4 inhibitor group (n = 57)** | | | |
| --- | --- | --- | --- |
|  | HR (95% CI) | *p* | Number of events |
| **OS** | 1.19 (0.22-6.44) | 0.860 | 16 |
| **PFS** | 0.36 (0.20-0.66) | 0.306 | 29 |
| **PD-1 inhibitor + TKI group (n = 19)** | | | |
|  | HR (95% CI) | *p* | Number of events |
| **OS** | 3.96 (2.85-5.51) | 0.169 | 7 |
| **PFS** | 2.88 (1.63-5.09) | 0.290 | 14 |
