# Supplementary material for: Paradoxical Effect of Myosteatosis on the Immune Checkpoint Inhibitor Response in Metastatic Renal Cell Carcinoma
Source: J Cachexia Sarcopenia Muscle. 2025 Mar 7;16(2):e13758. doi: 10.1002/jcsm.13758 (PMC11886412; doi:10.1002/jcsm.13758)
Supplement: Supplementary file 2 — Data S1 Supporting Information. [file JCSM-16-e13758-s004.docx]

S1. Escudier, B., et al., *CheckMate 025 Randomized Phase 3 Study: Outcomes by Key Baseline Factors and Prior Therapy for Nivolumab Versus Everolimus in Advanced Renal Cell Carcinoma.* Eur Urol, 2017. **72**(6): p. 962-971.

S2. Rini, B.I., et al., *Pembrolizumab plus Axitinib versus Sunitinib for Advanced Renal-Cell Carcinoma.* N Engl J Med, 2019. **380**(12): p. 1116-1127.

S3. Feng, S., et al., *Prognostic value of myosteatosis in patients with lung cancer: a systematic review and meta-analysis.* International Journal of Clinical Oncology, 2022. **27**(7): p. 1127-1138.

S4. Chen, B.-B., et al., *Sarcopenia and myosteatosis are associated with survival in patients receiving immunotherapy for advanced hepatocellular carcinoma.* European Radiology, 2023. **33**(1): p. 512-522.

S5. Martini, D.J., et al., *Body composition as an independent predictive and prognostic biomarker in advanced urothelial carcinoma patients treated with immune checkpoint inhibitors.* The oncologist, 2021. **26**(12): p. 1017-1025.

S6. Shen, W., et al., *Total body skeletal muscle and adipose tissue volumes: estimation from a single abdominal cross-sectional image.* J Appl Physiol (1985), 2004. **97**(6): p. 2333-8.
