# Supplementary figures and images for: Paradoxical Effect of Myosteatosis on the Immune Checkpoint Inhibitor Response in Metastatic Renal Cell Carcinoma
Source: J Cachexia Sarcopenia Muscle. 2025 Mar 7;16(2):e13758. doi: 10.1002/jcsm.13758 (PMC11886412; doi:10.1002/jcsm.13758)

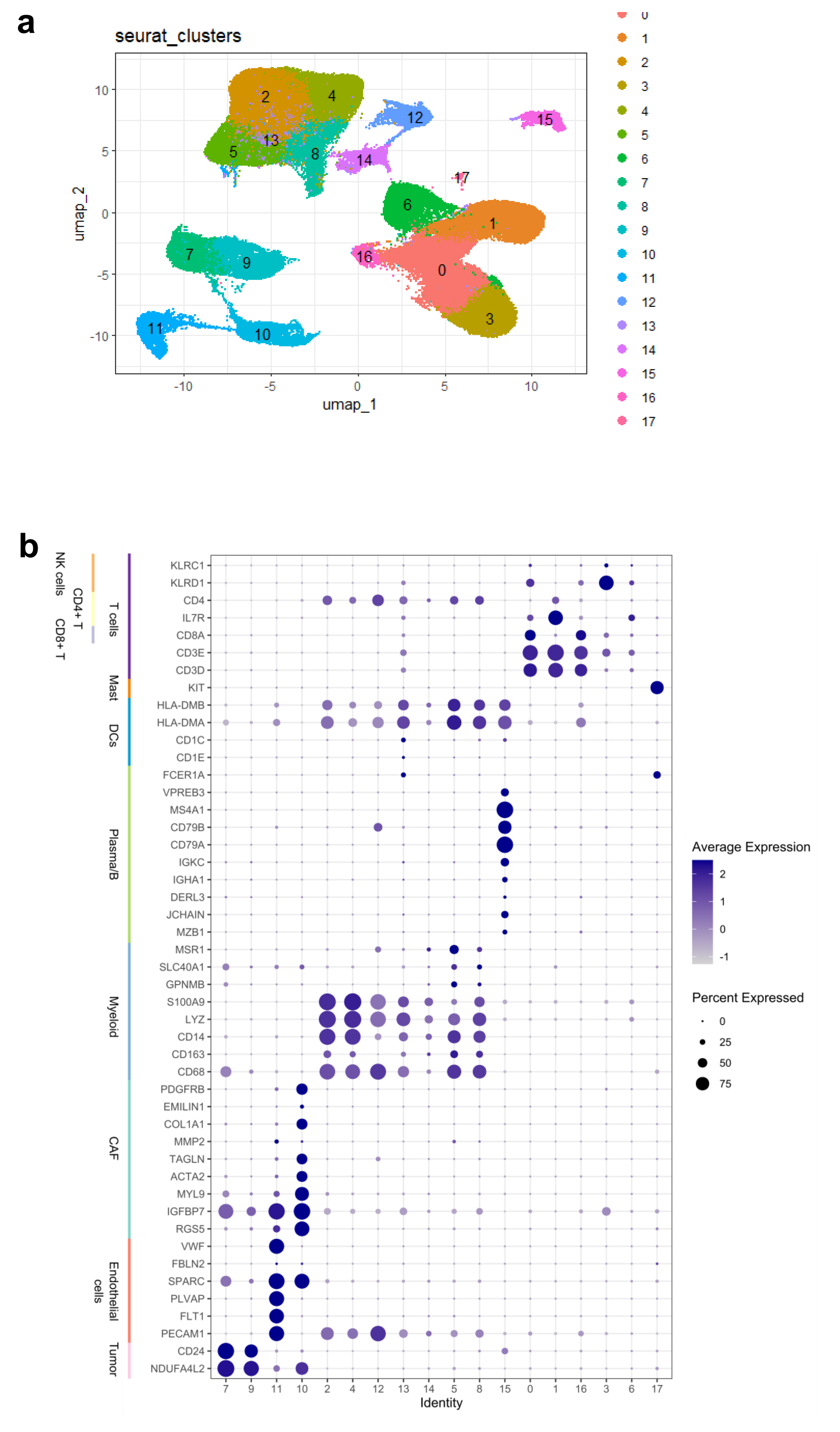

Supplement: Supplementary file 3 — Figure S1 Global cell type annotation. (a) UMAP plot of 92 457 cells from 12 patients with mRCC, coloured by 18 clusters. (b) Expression of marker genes for eight global cell types. Cluster 17 (169 cells) was removed because marker gene expression was not confirmed. [file JCSM-16-e13758-s005.tif]

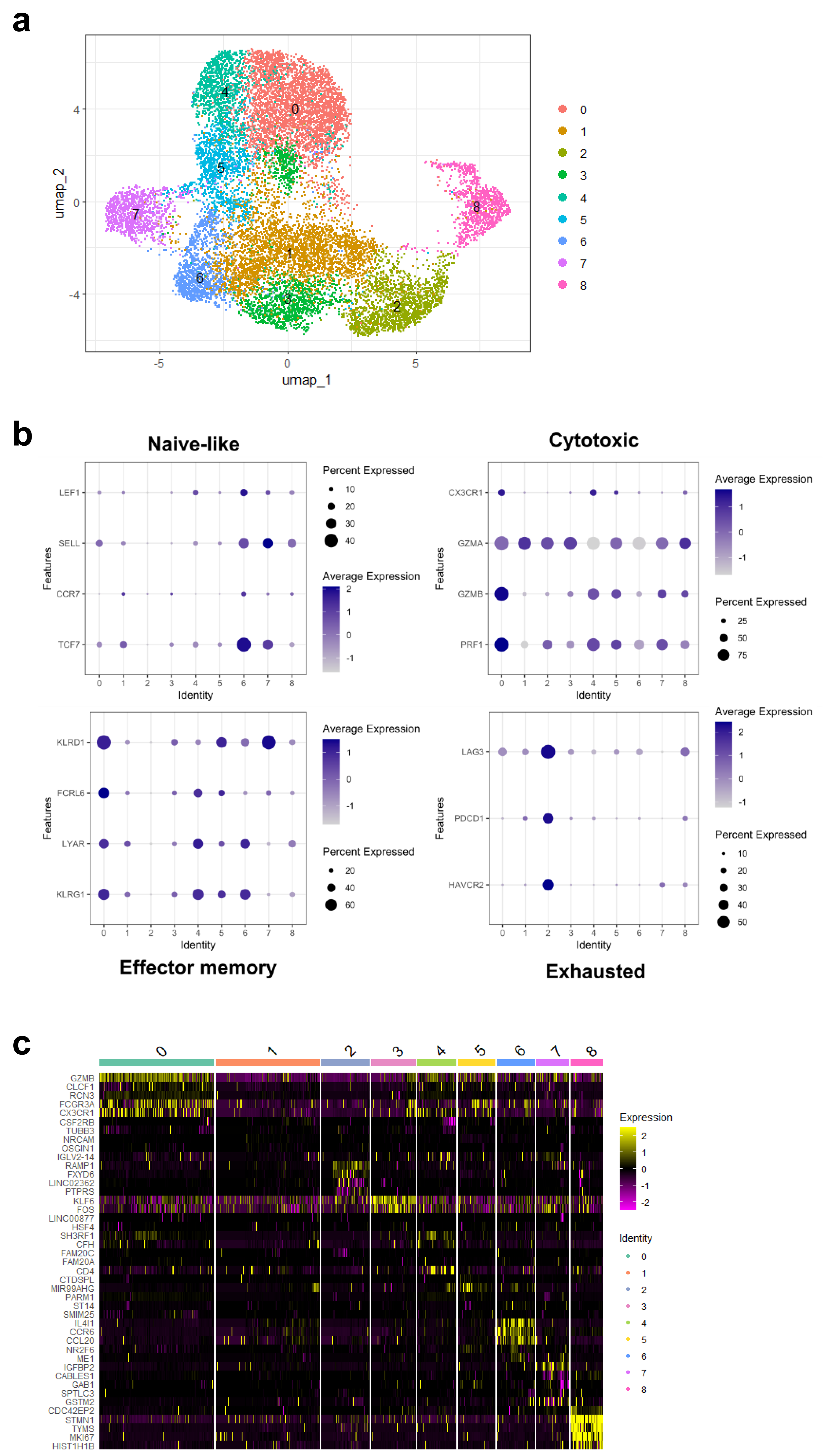

Supplement: Supplementary file 4 — Figure S2 CD8+ T‐cell sub‐clustering. (a) UMAP plot of CD8+ T cells from 12 patients with mRCC, coloured by nine clusters. (b) Dot plot showing marker gene expression in CD8+ T cells. (c) Heatmap showing the expression of the top five DEGs from each cluster in CD8+ T cells. [file JCSM-16-e13758-s001.tif]

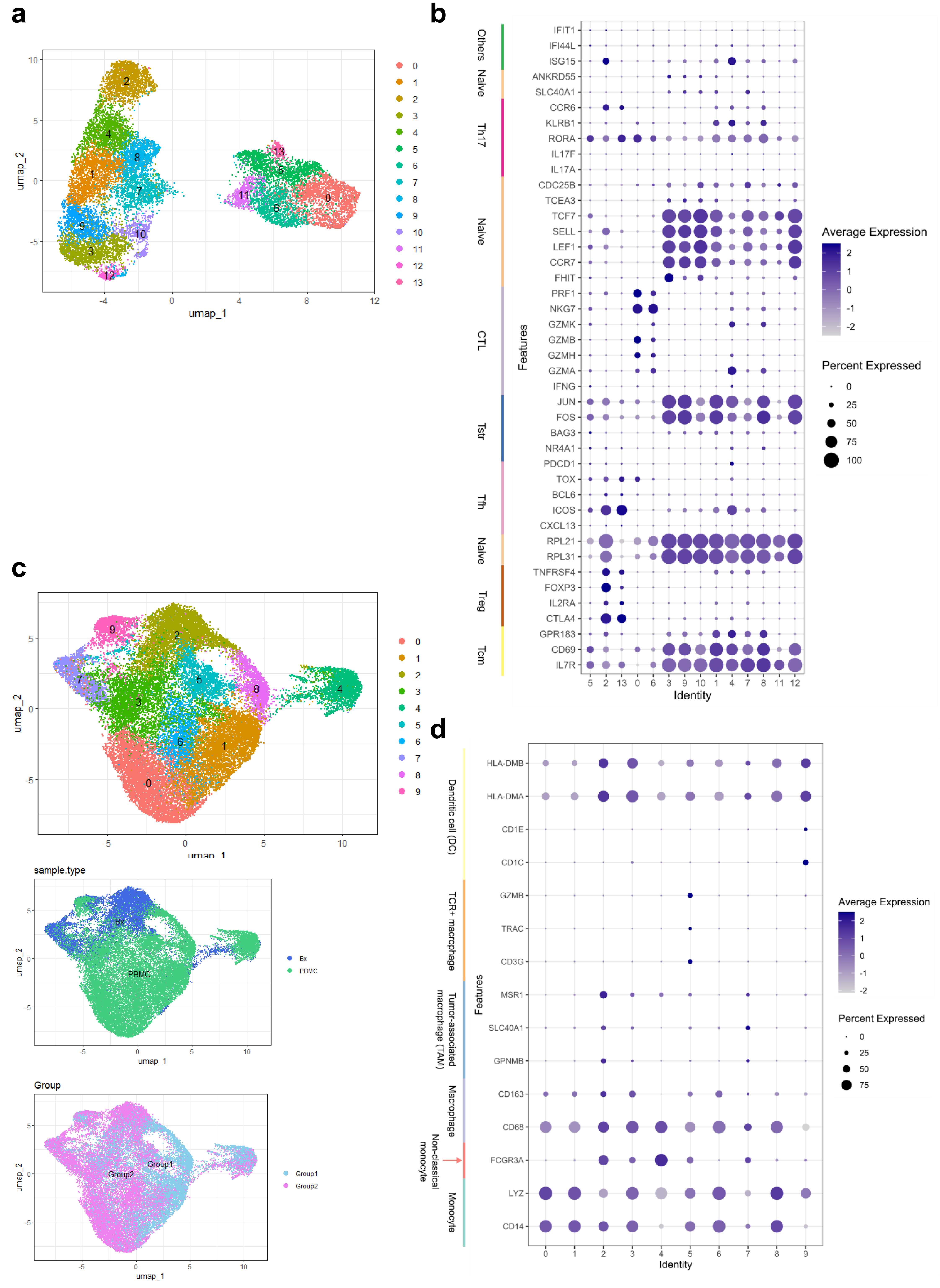

Supplement: Supplementary file 5 — Figure S3 Sub‐clustering of CD4+ T cells and myeloid cells. (a) UMAP plot of CD4+ T cells from 12 patients with mRCC, coloured by 14 clusters. (b) Expression of marker genes of CD4+ T subtypes. (c) UMAP plot of myeloid cells from 12 patients with mRCC, coloured by 10 clusters, sample type and myosteatosis group. (d) Expression of marker genes of myeloid cell subtypes. [file JCSM-16-e13758-s003.tif]

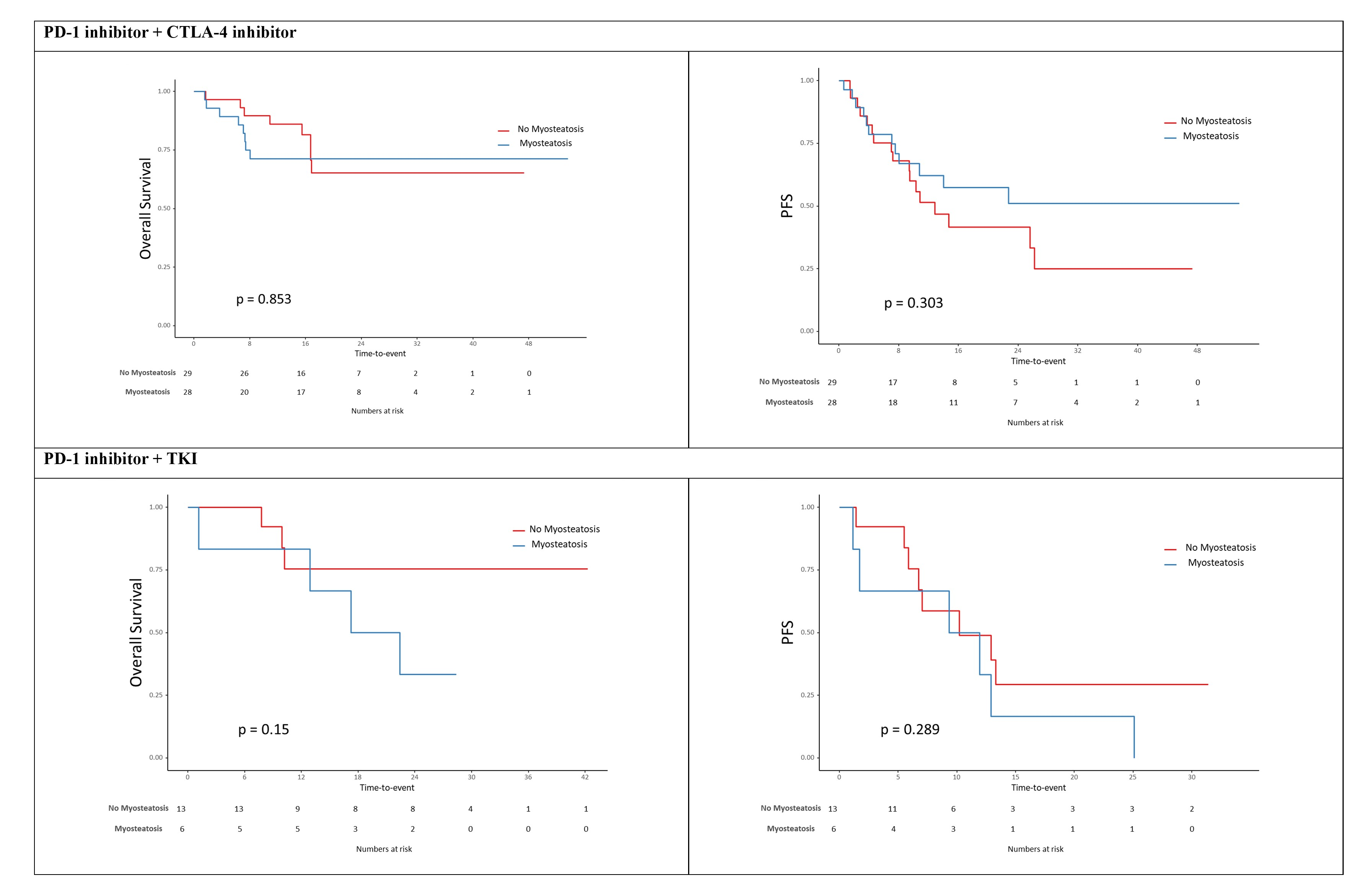

Supplement: Supplementary file 6 — Figure S4 Kaplan–Meier curve analysis of overall survival (OS) and progression‐free survival (PFS) according to treatment regimen and presence of myosteatosis in the clear cell RCC‐only cohort. [file JCSM-16-e13758-s006.tiff]
